# Prevalence and correlates of HIV infection among men who inject drugs in a remote area of Vietnam

**DOI:** 10.1186/s12954-018-0210-5

**Published:** 2018-02-14

**Authors:** Van T. Nghiem, Thanh C. Bui, Patrick P. Nadol, Son H. Phan, Binh T. Kieu, Ryan Kling, Theodore M. Hammett

**Affiliations:** 1 Department of Management, Policy and Community Health, University of Texas Health Science Center at Houston School of Public Health, Houston, TX 77030 USA; 2 Southwest Oncology Group Statistical Center, Fred Hutchinson Cancer Research Center, Seattle, WA 98109 USA; 3 Department of Family and Preventive Medicine, University of Oklahoma Health Sciences Center, Oklahoma City, OK 73104 USA; 4 Division of Global HIV/AIDS, U.S. Centers for Disease Control and Prevention, Hanoi, Vietnam; 5 International Health Division, Abt Associates, Bethesda, MD 20814 USA; 6 AIDS Healthcare Foundation, Hanoi, Vietnam; 7 U.S. Health Division, Abt Associates, Cambridge, MA 02138 USA; 8 Health Finance and Government Project, Abt Associates, Hanoi, Vietnam

**Keywords:** Vietnam, HIV prevalence (or infection), Men who inject drugs, Risk behaviors

## Abstract

**Background:**

Lack of information on the HIV epidemic among men who inject drugs (MWID) in northwestern Vietnam, a remote area, may hamper national efforts to control the disease. We examined HIV prevalence, needle–syringe sharing behaviors, and associated factors among MWID in three areas of northwestern Vietnam.

**Methods:**

We used descriptive analysis to report the characteristics, frequency of risk behaviors, and of access to healthcare services among the MWID. Univariable logistic regression was used to assess the associations between the HIV infection, needle–syringe sharing behaviors, and their independent variables. We further explored these associations in multivariable analyses where we included independent variables based on a priori knowledge and their associations with the dependent variables determined in univariable analyses (*p* <  0.25).

**Results:**

The HIV prevalence was 37.9, 16.9, and 18.5% for Tuan Giao, Bat Xat, and Lao Cai City, respectively, and 25.4% overall. MWID of Thai minority ethnicity were more likely to be HIV-positive (adjusted odds ratio (AOR) 3.55; 95% confidence interval (CI) 1.84–6.87). The rate of needle–syringe sharing in the previous 6 months was approximately 9% among the MWID in Tuan Giao and Lao Cai City, and 27.8% in Bat Xat. Two thirds of the participants never underwent HIV testing before this study. Ever having been tested for HIV before this study was not associated with any needle–syringe sharing behaviors. Among the HIV-positive MWID, those who received free clean needles and syringes were less likely to give used needles and syringes to peers (AOR 0.21; 95% CI 0.06–0.79). Going to a “hotspot” in the previous week was associated with increased odds of needle–syringe sharing in multiple subgroups.

**Conclusion:**

Our findings on HIV prevalence and testing participation among a subset of MWID in the northwestern Vietnam were corroborated with trend analysis results from the most recent HIV/STI Integrated Biological and Behavioral Surveillance report (data last collected in 2013.) We provided important insights into these MWID’s risky injection behaviors. We suggest heightened emphasis on HIV testing and needle and syringe provision for this population. Also, policymakers and program implementers should target hotspots as a main venue to tackle HIV epidemics.

## Background

In Vietnam, people who inject drugs comprised two thirds of the total human immunodeficiency virus (HIV) infections [1, 2]. Among that group, the vast majority (97%) are male [3–5]. Previous studies have shown multiple risk factors for HIV infection among people who inject drugs. One factor is receiving or giving used needles and/or syringes from other users [6–8]. Also, visiting shooting galleries or drug injection “hotspots” or injecting in public areas is associated with an increased likelihood of sharing injection equipment and of HIV transmission [9, 10]. Other risk factors for HIV infection in this group include age, education level, monthly income, and socioeconomic status [11–13].

Interventions to prevent and control HIV/acquired immune deficiency syndrome (AIDS), such as needle–syringe programs, peer education and outreach, and voluntary HIV testing, have been implemented throughout the country. However, these interventions have been focused in large cities (e.g., Hanoi and Ho Chi Minh City) where Kinh, the country’s majority ethnicity, populations are dense. Therefore, these interventions have primarily targeted the Kinh people. Minority people who inject drugs are disproportionately affected by poverty, lower education levels, and limited access to healthcare services, all of which put them at greater risk for HIV infection [14]. Des Jarlais and colleagues reported that minority people who inject drugs are twice more likely to be HIV positive than their majority ethnicity counterparts [15].

Ethnic minorities make up 14% of the Vietnamese population [16]. These minorities are concentrated in certain geographic areas that include the northwestern region. Men who inject drugs (MWID) in this region encounter challenges in accessing HIV/AIDS healthcare services, including limited communication materials in their dialects, low educational levels, limited HIV testing programs nearby, and difficulty in discreetly obtaining injection equipment [17]. The HIV/sexually transmitted infection (STI) Integrated Biological and Behavioral Survey (IBBS) report of 2011 (data collected in 2009) showed some descriptive statistics of HIV infection and the HIV-related risk behaviors among people living in the northwestern region who inject drugs [18]. This report, however, did not examine potential associations between drug users’ characteristics and their risk behaviors or HIV outcomes. In sum, very little is known about the prevalence of HIV, the prevalence of HIV-related risk behaviors, and associated factors among MWID in Vietnam’s northwestern area. This lack of understanding may hamper development of effective HIV interventions for ethnic minorities and thus could compromise the national effort for HIV/AIDS prevention and control.

Given these gaps in knowledge, our study aimed to (1) estimate HIV prevalence, (2) examine factors associated with HIV infection (e.g., demographic characteristics, drug injection behaviors, and visiting drug injection “hotspots”), and (3) investigate factors associated with needle–syringe sharing behaviors among a subset of MWID in the northwest.

## Methods

### Study design, sampling, and recruitment

We collected data from a cross-sectional survey from April to June 2011. Study sites included Tuan Giao district (Dien Bien province) and Bat Xat commune and Lao Cai City (Lao Cai province). These study sites were chosen because of high MWID prevalence, based on local reports and insight from our research team after multiple site visits. To recruit participants, we intended to apply a two-stage sampling process, known as time and location (i.e., “hotspot”) sampling. In our study, the term “hotspot” is preferred because it is from a Vietnamese term (*điểm nóng*) that is commonly used by MWID and is better understood by local HIV/AIDS researchers and staff. This method selected hotspots (locations) at a time that most MWID would be present and then implemented a random selection of MWID for each hotspot. However, a widespread anti-drug campaign before and during the time of data collection led to detention and concealment of many MWID. Therefore, we recruited all eligible participants at targeted hotspots and then used a chain referral method for further recruitment (i.e., all recruited participants were asked to refer other potential participants to the study). We continued the recruitment process until we reached the desired sample size. Eligibility criteria were being male, being 18 years old or older, and having last injected drugs within 1 month before the data collection. We focused on the MWID because, according to the local reports, very few females injected drugs in the region.

### Procedure

At the selected hotspots, we screened the eligibility of potential participants and, if eligible, invited them to the designated data collection sites at a scheduled time. Those who participated in the study could refer other eligible MWID. Referred participants were instructed to contact the research team (via telephone or in person) to schedule the interview and blood test. When participants arrived at the collection site, research team members again checked participants’ eligibility by asking screening questions about their drug injection behavior or inspecting the injection track marks. In the presence of a team member witness, a researcher read the description of the study, explained procedure details, and obtained oral informed consent from the participant. The research team had translators to communicate with the participants who did not speak the Kinh language. The Institutional Review Board of Abt Associates and the Ethics Committee of Centers for Disease Control and Prevention in the USA, and Hanoi School of Public Health’s Ethical Review Board in Vietnam approved the study’s research protocol and questionnaires. All of these boards approved oral informed consent because of potential severe consequences for participants if there were a confidentiality breach associated with a written consent.

After informed consent was obtained, a trained researcher used a structured questionnaire to conduct a 30- to 45-min, face-to-face interview in a private room with each participant. Responses were recorded on paper and were entered into a computer-based statistical program. After the interview, the participant was provided with pre-test counseling. Then, the participant’s blood was drawn by a trained phlebotomist from a local health center for HIV testing. Each participant received 50,000 Vietnamese Dong (~ 2.40 US dollars) in compensation for his time and transportation. We transported the blood samples to provincial HIV/AIDS center laboratories for testing, according to national guidelines. Determine™ HIV-1/2 (AbbottLaboratories®, Tokyo, Japan) was used for rapid testing; two enzyme immunoassay tests including Genscreen™ HIV–1/2 version 2 (Bio-Rad Laboratories, CA, USA) and Murex HIV 1.2.0 (Murex Biotech, Dartford, UK) for confirmation testing. The participants were advised to contact designated local staff for post-test counseling, test results, and appropriate referrals (e.g., outpatient treatment clinics). This testing procedure took approximately 4 weeks.

### Measures

Outcomes were HIV positivity and needle–syringe sharing behaviors. HIV positivity was diagnosed by the above-described blood testing. Needle–syringe sharing behaviors were obtained through self-report. These behaviors, including giving and receiving injection equipment, were queried for two different time frames: last 6 months and last month prior to the study.

Independent variables used to evaluate HIV positivity were demographic characteristics and lifetime behaviors. These included residential area (i.e., study site), age, ethnicity, education, duration of injection drug use, and receiving or giving used needles and/or syringes in the participant’s lifetime. Independent variables for needle–syringe sharing behaviors included demographic characteristics and relevant factors suggested by previous studies. These were study site, age, ethnicity, education, duration of injection drug use, frequency of injection drug use in the previous month, going to a hotspot in the previous week, and receiving free clean needles and syringes in the previous 6 months. All behavioral measures (e.g., for drug use and sexual activities) were adapted from internationally standardized questionnaires [19].

We tested the potential associations of several risk factors with different needle–syringe sharing behaviors based on their potential implications for future behavioral interventions. We first examined the factors associated with receiving or giving used needles and/or syringes to gain an overall understanding of risk factors for those behaviors. We then separately examined the preventable behavior of receiving used needles and/or syringes and its associated factors among HIV-negative MWID because this behavior would place those men at risk for HIV infection and could subsequently increase HIV incidence. Finally, we investigated the behavior of giving used needles and/or syringes and its associated factors among HIV-positive MWID because this practice could transmit the virus to HIV-negative MWID. We examined the behavior of giving and the behavior of receiving separately because interventions to modify these two behaviors target different populations and require different theoretical constructs.

### Analysis

We first explored sociodemographic and behavioral characteristics of the study population across the three study sites. Univariable logistic regression was used to assess the associations between the dependent and independent variables. We then performed multivariable logistic regression models to provide adjusted association estimates. Independent variables were selected for inclusion into multivariable models based on a priori knowledge and their potential associations with the dependent variables in univariable analyses (*p* value < 0.25, as suggested by Hosmer and Lemeshow) [20]. Significance level was determined at 0.05. We used SAS 9.4 software (SAS, Cary, NC) to perform all analyses.

## Results

### Sample characteristics

Seven hundred eighty-five MWID were recruited into the study. Those from Bat Xat were the most ethnically diverse, and those in Lao Cai City were primarily of Kinh ethnicity (91.3%) (Table 1). Sixty-five percent of the participants in Tuan Giao were of Thai ethnicity. The frequency of injection drug use in the previous month was substantially different across the study sites. We found approximately 9% of the participants in Tuan Giao or Lao Cai City giving or receiving used needles and/or syringes in the previous 6 months, 27.8% in Bat Xat. Lao Cai City had the most participants going to hotspots in the previous week (80.2%), compared with Bat Xat (61.0%) and in Tuan Giao (34.4%). More than half (58.2%) of the participants reported having no sex partners in the previous 6 months. Among those who had one or more sex partners, only 70 participants responded to questions on condom use with irregular sexual partners. Therefore, we did not examine sexual behaviors in the subsequent analyses. Almost two thirds of the participants had not been tested for HIV before this study, Tuan Giao participants having the highest rate of not having been tested for HIV (58.7%).

**Table 1:** Sociodemographic and behavioral characteristics of men who injected drugs in northwestern Vietnam

Characteristics	Total, *n* (%)	Tuan Giao, *n* (%)	Bat Xat, *n* (%)	Lao Cai City, *n* (%)	*p* value^a^
Total	785 (100.0)	292 (37.2)	195 (24.9)	297 (37.9)	–
Demographics and lifetime behaviors					
Age groups					< 0.001
18–28	206 (26.3)	97 (33.2)	30 (15.4)	79 (26.6)	
29–35	214 (27.3)	87 (29.8)	46 (23.6)	81 (27.3)	
36–41	168 (21.3)	51 (17.5)	45 (23.1)	71 (23.9)	
42 or older	197 (25.1)	57 (19.5)	74 (38.0)	66 (22.2)	
Education level					< 0.001
No education	116 (14.8)	36 (12.3)	77 (39.5)	3 (1.0)	
Grades 1–9	446 (56.8)	204 (69.9)	93 (47.7)	148 (49.8)	
Grade 10 or higher	223 (28.4)	52 (17.8)	25 (12.8)	146 (49.2)	
Ethnicity					< 0.001
Kinh	388 (49.4)	59 (20.2)	58 (29.7)	271 (91.3)	
Thai	194 (24.7)	192 (65.5)	2 (1.0)	0 (0.0)	
H’mong	54 (6.9)	3 (1.0)	51 (26.2)	0 (0.0)	
Dao	39 (5.0)	0 (0.0)	39 (20.0)	0 (0.0)	
Others	110 (14.0)	38 (13.0)	45 (23.1)	26 (8.8)	
Duration of injection drug use					
≤ 1 year	246 (31.4)	108 (37.0)	77 (39.5)	61 (20.5)	< 0.001
2–3 years	214 (27.2)	79 (27.1)	68 (34.9)	66 (22.2)	
4–6 years	138 (17.6)	58 (19.9)	20 (10.3)	60 (20.2)	
> 6 years	187 (23.9)	47 (16.1)	30 (15.4)	110 (37.0)	
Ever gave or received used needles and/or syringes					< 0.001
No	630 (80.3)	254 (87.0)	128 (66.0)	247 (83.2)	
Yes	154 (19.7)	38 (13.0)	66 (34.0)	50 (16.8)	
Recent behaviors					
Gave or received used needles and/or syringes in the previous 6 months					< 0.001
No	676 (86.3)	266 (91.1)	140 (72.2)	270 (90.9)	
Yes	107 (13.7)	26 (8.9)	54 (27.8)	27 (9.1)	
Frequency of injection drug use in the previous month					< 0.001
≤ 1 time/day	300 (38.4)	61 (21.0)	120 (61.5)	119 (40.2)	
≥ 2 times/day	483 (61.6)	230 (79.0)	75 (38.5)	177 (59.8)	
Gave used needles and/or syringes in the previous month					< 0.001
No	672 (91.2)	262 (93.6)	155 (85.2)	254 (92.7)	
Yes	65 (8.8)	18 (6.4)	27 (14.8)	20 (7.3)	
Received used needles and/or syringes in the previous month					< 0.001
No	652 (88.5)	260 (92.9)	139 (76.4)	252 (92.0)	
Yes	85 (11.6)	20 (7.1)	43 (23.6)	22 (8.0)	
Went to a hotspot in the previous week					< 0.001
No	299 (43.1)	187 (65.6)	62 (39.0)	49 (19.8)	
Yes	393 (56.9)	98 (34.4)	97 (61.0)	198 (80.2)	
Received free clean needles and syringes in the previous 6 months					< 0.001
No	378 (48.2)	134 (45.9)	140 (71.8)	104 (35.0)	
Yes	407 (51.8)	158 (54.1)	55 (28.2)	193 (65.0)	
Number of sex partners in the previous 6 months					< 0.001
1	234 (30.2)	143 (49.0)	125 (64.1)	181 (63.7)	
0	449 (58.2)	119 (40.8)	51 (26.2)	63 (22.2)	
Multiple	89 (11.5)	30 (10.3)	19 (9.7)	40 (14.1)	
HIV status					< 0.001
HIV testing before this study					
Ever tested and HIV+	80 (11.7)	43 (16.9)	12 (9.0)	25 (8.6)	
Ever tested and HIV−	166 (24.4)	62 (24.4)	19 (14.2)	84 (28.9)	
Never tested	434 (63.8)	149 (58.7)	103 (76.9)	182 (62.5)	
HIV prevalence (results from blood test for this study)	199 (25.4)	111 (37.9)	33 (16.9)	55 (18.5)	< 0.001

^a^*p* values for proportion comparison in each characteristics across three study sites

### HIV prevalence and associated factors

The overall HIV prevalence was 25.4%; 37.9, 16.9, and 18.5% for Tuan Giao, Bat Xat, and Lao Cai City, respectively. Among the HIV-positive MWID, 59.8% had never been tested before this study. In addition, of those who had never been tested, 23% were HIV positive.

Age, ethnicity, and duration of injection drug use were associated with HIV positivity both in univariate analyses and in the adjusted analysis (Table 2). Compared with the youngest age group (18–28 years), those 29–35 years old were more likely to be HIV positive (adjusted odds ratio [AOR] = 1.62, 95% confidence interval [CI] 1.10–2.60), yet participants in the oldest age group (≥ 42 years) were less likely to be HIV positive (AOR = 0.46, 95% CI 0.25–0.83). Participants of Thai ethnicity had higher odds of being HIV positive (AOR = 3.55, 95% CI 1.84–6.87) than the Kinh. The MWID with longer duration of injection drug use had higher odds of being HIV positive. We did not examine recent behaviors in the multivariable analysis because temporal relationships between these behaviors and HIV positivity could not be determined; thus, the results would not be interpretable.

**Table 2:** Associations between HIV infection and sociodemographic or behavioral characteristics among men who injected drugs in northwestern Vietnam

Characteristics	HIV positivity				
	*n* (%^a^)	Unadjusted OR (95% CI)	*p* value	Adjusted OR (95% CI)^b^	*p* value
Study sites					
Tuan Giao	111 (38.0)	1		1	
Bat Xat	33 (16.9)	*0.33 (0.26–0.52)*	*< 0.001*	1.44 (0.75–2.79)	0.278
Lao Cai City	55 (18.5)	*0.37 (0.26–0.54)*	*< 0.001*	0.74 (0.40–1.36)	0.329
Demographics and lifetime behaviors					
Age groups					
18–28	52 (25.2)	1		1	
29–35	80 (37.4)	*1.77 (1.16–2.69)*	*0.008*	*1.62 (1.10–2.60)*	*0.046*
36–41	37 (22.0)	0.84 (0.52–1.35)	0.467	0.64 (0.36–1.12)	0.116
42 or older	30 (15.2)	*0.53 (0.32–0.88)*	*0.013*	*0.46 (0.25–0.83)*	*0.010*
Ethnicity					
Kinh	81 (20.9)	1			
Thai	90 (46.4)	*3.28 (2.26–4.77)*	*< 0.001*	*3.55 (1.84–6.87)*	*< 0.001*
H’mong	1 (1.9)	*0.07 (0.01–0.53)*	*0.010*	*0.07 (0.01–0.61)*	*0.016*
Dao	3 (7.7)	0.316 (0.095–1.052)	0.060	0.34 (0.08–1.42)	0.140
Others	24 (21.8)	1.058 (0.632–1.769)	0.831	0.89 (0.48–1.64)	0.707
Education level					
No education	22 (19.0)	1		1	
Grades 1–9	141 (31.6)	*1.98 (1.19–3.27)*	*0.008*	0.99 (0.50–1.95)	0.969
Grade 10 or higher	36 (16.1)	0.82 (0.458–1.48)	0.513	0.46 (0.21–1.02)	0.055
Duration of injection drug use					
≤ 1 year	38 (15.5)	1		1	
2–3 years	51 (23.8)	*1.71 (1.07–2.73)*	*0.024*	*2.15 (1.26–3.65)*	*0.005*
4–6 years	50 (36.2)	*3.11 (1.91–5.08)*	*< 0.001*	*4.36 (2.46–7.73)*	*< 0.001*
> 6 years	60 (32.1)	*2.59 (1.63–4.11)*	*< 0.001*	*5.63 (3.10–10.26)*	*< 0.001*
Ever gave or received used needles and/or syringes					
No	163 (25.9)	1			
Yes	36 (23.4)	0.87 (0.58–1.32)	0.524		
Recent behaviors					
Frequency of injection drug use in the previous month				–	
≤ 1 time/day	59 (19.7)	1		–	
≥ 2 times/day	140 (29.0)	*1.67 (1.18–2.36)*	*0.004*	–	
Gave or received used needles and/or syringes in the previous 6 months				–	
No	182 (26.9)	1			
Yes	17 (15.9)	*0.51 (0.30–0.89)*	*0.017*		
Gave used needles and/or syringes in the previous month				–	
No	166 (24.7)	1		–	
Yes	14 (21.5)	0.84 (0.45–1.55)	0.571	–	
Received used needles and/or syringes in the previous month				–	
No	164 (25.2)	1		–	
Yes	16 (18.8)	0.69 (0.39–1.22)	0.204	–	
Went to a hotspot in the previous week				–	
No	101 (33.8)	1		–	
Yes	76 (19.3)	*0.47 (0.33–0.67)*	*< 0.001*	–	
Received free clean needles and syringes in the previous 6 months				–	
No	90 (23.8)	1		–	
Yes	109 (26.8)	1.17 (0.85–1.62)	0.339	–	
Number of sex partners in the previous 6 months				–	
1	67 (28.6)	1		–	
0	116 (25.8)	0.87 (0.61–1.24)	0.434	–	
Multiple	14 (15.7)	*0.47 (0.25–0.88)*	*0.019*	–	

*OR* odds ratio, *CI* confidence interval

^a^Percent by *n* in each category of sociodemographic or behavioral characteristics

^b^In the multivariable model, there were 199 HIV-positive and 586 HIV-negative participants

### Needle–syringe sharing behaviors among MWID

Table 3 shows factors associated with needle–syringe sharing (receiving or giving used needles, and/or syringes or both) in the previous 6 months among the MWID who had never been tested for HIV before our study. In the adjusted analysis, older participants were less likely to share needle–syringe in the previous 6 months than were 18- to 28-year-olds. However, the oldest participants (≥ 42 years) had similar needle–syringe sharing behaviors in the previous 6 months as the youngest group. Compared with participants who injected drugs for ≤ 1 year, those who had injected for a longer time (2–3 years and 4–6 years) were more likely to have received or given used needles and/or syringes in the previous 6 months (AOR range 2.62–4.29). Going to a hotspot in the previous week was associated with increased odds of receiving or giving used needles and syringes in the previous 6 months (AOR = 2.81, 95% CI 1.34–5.90).

**Table 3:** Factors associated with receiving or giving used needles and/or syringes in the previous 6 months among men who injected drugs in northwestern Vietnam and had never been tested for HIV before this study

Characteristics		Received or gave used needles and/or syringes in the previous 6 months			
	*n* (%^a^)	Unadjusted OR (95% CI)	*p* value (for trend)	Adjusted OR^b^ (95% CI)	*p* value (for trend)
Study sites					
Tuan Giao	144 (38.6)	1		1	
Bat Xat	82 (22.0)	*2.41 (1.21–4.79)*	*0.012*	1.22 (0.43–3.44)	0.706
Lao Cai City	147 (39.4)	0.69 (0.33–1.44)	0.325	0.58 (0.18–1.92)	0.373
Age groups			(0.345)		(0.060)
18–28	92 (24.7)	1		1	
29–35	109 (29.2)	0.66 (0.31–1.39)	0.271	*0.40 (0.17–0.95)*	*0.037*
36–41	89 (23.9)	0.46 (0.20–1.09)	0.079	*0.25 (0.09–0.68)*	*0.007*
42 or older	83 (22.3)	0.76 (0.35–1.67)	0.500	0.45 (0.17–1.17)	0.101
Ethnicity					
Kinh	173 (46.4)	1		1	
Thai	96 (25.7)	1.14 (0.50–2.47)	0.790	1.12 (0.31–4.13)	0.862
H’mong	16 (4.3)	*3.91 (1.22–12.54)*	*0.022*	4.07 (0.83–20.06)	0.085
Dao	21 (5.6)	*4.31 (1.54–12.06)*	*0.006*	4.59 (0.96–21.89)	0.056
Others	67 (18.0)	*2.28 (1.06–4.89)*	*0.035*	2.25 (0.84–6.05)	0.108
Education level			(0.014)		(0.370)
No education	43 (11.5)	1		1	
Grades 1–9	224 (60.1)	0.52 (0.24–1.13)	0.099	1.06 (0.38–2.94)	0.912
Grade 10 or higher	106 (28.4)	*0.30 (0.12–0.78)*	*0.013*	0.79 (0.22–2.81)	0.712
Duration of injection drug use			(0.634)		(0.061)
≤ 1 year	126 (33.8)	1		1	
2–3 years	100 (26.8)	2.04 (0.95–4.36)	0.067	*2.62 (1.09–6.30)*	*0.032*
4–6 years	71 (19.0)	*2.33 (1.04–5.23)*	*0.041*	*4.29 (1.65–11.19)*	*0.003*
> 6 years	76 (20.4)	1.02 (0.40–2.59)	0.962	2.68 (0.85–8.47)	0.093
Frequency of injection drug use in the previous month					
≤ 1 time/day	141 (37.8)	1		1	
≥ 2 times/day	232 (62.2)	0.75 (0.42–1.34)	0.335	0.77 (0.38–1.55)	0.457
Went to a hotspot in the previous week					
No	155 (41.6)	1		1	
Yes	218 (58.5)	*2.10 (1.13–3.95)*	*0.022*	*2.81 (1.34–5.90)*	*0.006*
Received free clean needles and syringes in the previous 6 months					
No	186 (49.9)	1		1	
Yes	187 (50.1)	0.57 (0.32–1.01)	0.057	0.63 (0.31–1.28)	0.201

*OR* odds ratio, *CI* confidence interval

^a^Percent by *n* in each category of sociodemographic or behavioral characteristics

^b^In the multivariable model, there were 55 participants who received used needles and/or syringes and 318 participants who did not

Table 4 displays factors associated with receiving used needles and/or syringe in the previous month among HIV-negative participants. In the adjusted model, those who went to a hotspot in the previous week were more likely to receive used needles and/or syringes (AOR = 3.03; 95% CI 1.28–7.19).

**Table 4:** Factors associated with receiving used needles and/or syringes in the previous month among HIV-negative men who injected drugs in northwestern Vietnam

Characteristics		Received used needles and/or syringes in the previous month			
	*n* (%^a^)	Unadjusted OR (95% CI)	*p* value (for trend)	Adjusted OR^b^ (95% CI)	*p* value (for trend)
Study sites					
Tuan Giao	146 (35.8)	1		1	
Bat Xat	79 (19.4)	*3.12 (1.37–7.11)*	*0.007*	1.70 (0.47–6.17)	0.417
Lao Cai City	182 (44.7)	1.02 (0.45–2.33)	0.957	1.13 (0.33–3.94)	0.848
Age groups			(0.458)		
18–28	104 (25.6)	1		–	
29–35	97 (23.8)	1.08 (0.43–2.72)	0.870	–	
36–41	101 (24.8)	0.81 (0.31–2.14)	0.669	–	
42 or older	105 (25.8)	1.33 (0.56–3.18)	0.524	–	
Ethnicity					
Kinh	233 (57.3)	1		1	
Thai	77 (18.9)	1.45 (0.57–3.71)	0.434	2.91 (0.73–11.51)	0.129
H’mong	16 (3.9)	*6.61 (2.03–21.49)*	*0.002*	5.19 (0.89–30.41)	0.068
Dao	20 (4.9)	*4.84 (1.55–15.14)*	*0.007*	3.18 (0.56–18.06)	0.191
Others	61 (15.0)	*2.52 (1.04–6.06)*	*0.040*	*3.01 (1.08–8.38)*	*0.035*
Education level			(0.002)		(0.189)
No education	38 (9.3)	1		1	
Grades 1–9	223 (54.8)	*0.31 (0.13–0.71)*	*0.006*	0.66 (0.21–2.09)	0.484
Grade 10 or higher	146 (35.9)	*0.18 (0.07–0.49)*	*0.001*	0.51 (0.13–2.02)	0.336
Duration of injection drug use			(0.383)		(0.059)
≤ 1 year	142 (34.9)	1		1	
2–3 years	102 (25.1)	2.16 (0.89–5.26)	0.091	2.13 (0.78–5.81)	0.142
4–6 years	67 (16.5)	*2.90 (1.14–7.39)*	*0.025*	*3.83 (1.32–11.13)*	*0.014*
> 6 years	96 (23.6)	1.34 (0.50–3.61)	0.559	2.52 (0.78–8.19)	0.125
Frequency of injection drug use in the previous month					
≤ 1 time/day	152 (37.4)	1		1	
≥ 2 times/day	255 (62.7)	0.66 (0.35–1.27)	0.211	0.69 (0.31–1.51)	0.347
Went to a hotspot in the previous week					
No	155 (38.1)	1		1	
Yes	252 (61.9)	*2.36 (1.09–5.09)*	*0.029*	*3.03 (1.28–7.19)*	*0.012*
Received free clean needles and syringes in the previous 6 months					
No	187 (46.0)	1		–	
Yes	220 (54.1)	0.79 (0.41–1.51)	0.476	–	
Ever tested for HIV					
No	265 (65.1)	1		1	
Yes	142 (34.9)	0.57 (0.27–1.20)	0.141	0.93 (0.40–2.13)	0.862

*OR* odds ratio, *CI* confidence interval

^a^Percent by *n* in each category of sociodemographic or behavioral characteristics

^b^In the multivariable model, there were 41 participants who received used needles and/or syringes and 366 participants who did not

Table 5 reports the factors associated with giving used needles and/or syringes in the previous month among the HIV-positive MWID. Going to a hotspot in the previous week increased the odds of giving used needles and/or syringes in the previous month (AOR = 4.57; 95% CI 1.08–19.26). Meanwhile, receiving free clean needles and syringes was associated with reduced odds of giving used needles and/or syringes (AOR = 0.21; 95% CI 0.06–0.79).

**Table 5:** Factors associated with giving used needles and/or syringes in the previous month among HIV-positive men who injected drugs in northwestern Vietnam

		Gave used needles and/or syringes in the previous month			
Variables	*n* (%)^a^	Unadjusted OR (95% CI)	*p* value (for trend)	Adjusted OR^b^ (95% CI)	*p* value
Study sites					
Tuan Giao	104 (57.8)	1		1	
Bat Xat	28 (15.6)	1.52 (0.28–8.30)	0.627	0.37 (0.04–3.72)	0.395
Lao Cai City	48 (26.7)	*3.38 (1.01–11.27)*	*0.047*	2.75 (0.72–10.56)	0.140
Age groups			(0.120)		
18–28	49 (27.2)	1		–	
29–35	72 (40.0)	0.54 (0.15–1.86)	0.325	–	
36–41	33 (18.3)	0.46 (0.09–2.45)	0.364	–	
42 or older	26 (14.4)	0.29 (0.03–2.52)	0.260	–	
Ethnicity					
Kinh	70 (38.9)	1		–	
Thai	85 (47.2)	0.22 (0.04–1.08)	0.062	–	
H’mong	1 (0.6)	–^c^	–	–	
Dao	3 (1.7)	–^c^	–	–	
Others	21 (11.7)	1.50 (0.35–6.40)	0.584	–	
Education level			(0.623)		
No education	20 (11.1)	1		–	
Grades 1–9	130 (72.2)	0.83 (0.17–4.06)	0.820	–	
Grade 10 or higher	30 (16.7)	0.31 (0.03–3.68)	0.354	–	
Duration of injection drug use			(0.194)		
≤ 1 year	37 (20.6)	1		–	
2–3 years	45 (25.0)	0.81 (0.11–6.08)	0.841	–	
4–6 years	47 (26.1)	2.08 (0.38–11.41)	0.398	–	
> 6 years	51 (28.3)	1.90 (0.35–10.39)	0.458	–	
Frequency of injection drug use					
≤ 1 time/day	52 (28.9)	1		–	
≥ 2 times/day	128 (71.1)	1.54 (0.41–5.75)	0.524	–	
Went to a hotspot in the previous week					
No	65 (40.6)	1		1	
Yes	95 (59.4)	*5.58 (1.47–21.14)*	*0.012*	*4.57 (1.08–19.26)*	*0.039*
Received free clean needles and syringes in the previous 6 months					
No	100 (55.6)	1		1	
Yes	80 (44.4)	*0.29 (0.09–0.97)*	*0.044*	*0.21 (0.06–0.79)*	*0.021*
Ever tested for HIV					
No	98 (58.7)	1		–	
Yes	69 (41.3)	0.61 (0.18–2.06)	0.425	–	

*OR* odds ratio, *CI* confidence interval

^a^Percent by *n* in each category of sociodemographic or behavioral characteristics

^b^In the multivariable model, there were 13 participants who gave used needles and/or syringes and 146 participants who did not

^c^Could not be computed because the number in this subgroup who injected drugs and gave used needles and syringes in the previous month was small or none

Overall, we saw several consistent needle–syringe sharing behavioral patterns. The frequency of injection drug use and ever having been tested for HIV before our study were not associated with any of the needle–syringe sharing behaviors examined (Tables 3, 4, and 5). Lastly, there was no significant difference in the sharing behaviors across the four ethnic groups—Kinh, Thai, H’mong, and Dao.

## Discussion

To our best knowledge, this study is among only a few studies that examined the prevalence of HIV infection among the MWID in the northwestern area of Vietnam, and this is the first study to examine correlates of HIV infection and of needle–syringe sharing behaviors in this population. We found three key points: first, HIV prevalence among MWID was significantly different across ethnicities. Second, a large number of the MWID had not been tested for HIV before this study, and the HIV-positive MWID who received free clean needles and syringes were less likely to give their used needles and syringes to peers. Third, going to a hotspot in the previous week emerged as a salient factor associated with the needle–syringe sharing behaviors.

To the first point, Thai ethnicity MWID had a significantly higher prevalence of infection than the other groups. On the one hand, further research is needed to investigate why there are such health disparities. This type of research could help reduce HIV incidence and ensure that similar disparities do not exist in the subsequent HIV/AIDS care continuum. On another side, prevention efforts should focus on maintaining HIV-negative status among MWID of other ethnicities with low HIV prevalence (including Kinh, H’mong, and Dao). Additionally, it is imperative to design and conduct effective prevention programs that suit this ethnically diverse area. For example, health promotion messages should be culturally and linguistically appropriate [21]. Bui and colleagues found that residents of areas with several different ethnicities had low awareness of HIV [22]. If this low awareness is also true for MWID in our study area, there would be demand for interventions to improve HIV-related awareness, knowledge, and attitudes.

We compared our results for Tuan Giao with the results from the most recent HIV/sexually transmitted infections (STI) IBBS report in Vietnam [23]. This IBBS report provided findings from the data collected in 2013 and trend analyses comprising the findings from the data collected in 2005 and 2009. Of note, this report provided aggregated data for the people who injected drugs from three study sites in Dien Bien province, one of which was Tuan Giao. In our study, we found that HIV prevalence in Tuan Giao was 37.9%; this is consistent with the IBBS’s decreasing HIV trend for Dien Bien province, from 57% in 2006 to 31% in 2013. Our study also corroborated IBBS’s statistics with respect to HIV testing. In Dien Bien province, HIV testing rates increased from 25% in 2009 to 48% in 2013. In Tuan Giao, we found the 2011 rate of ever having had an HIV test was 41.3%.

The rates of ever having been tested for HIV before this study in Bat Xat (23.1%) and Lao Cai (37.5%) were lower than the median HIV testing rate (38%) from a 10-province survey of MWID conducted in 2009–2010 [24]. Of note, many of these 10 provinces (including Hanoi, Hai Phong, Quang Ninh, Nghe An, and Ho Chi Minh City) had received technical and financial support from large donors for HIV/AIDS control programs (e.g., The US President’s Emergency Plan for HIV/AIDS Relief and The Global Fund to Fight AIDS, Tuberculosis, and Malaria). This support might have contributed to better HIV testing rates. The rate of MWID in Tuan Giao who had ever been tested for HIV before our study was 41.3%, the highest among our three study sites; however, this rate was still lower than rates seen in several of the 10 provinces surveyed [24]. By contrast, in 2009, 80% of people who injected drugs from 20 cities in the USA reported to have had at least one HIV test during their lifetime [25]. In addition, people who inject drugs in the USA are recommended to have HIV screening annually or more often [26].

Given the low rates of HIV testing among our participants, it is imperative for policymakers to focus resources to improve HIV testing uptake among MWID. Previous studies have demonstrated that HIV testing programs were highly cost-effective in that they averted a large number of HIV infections thereby reducing the economic burden associated with these infections [27, 28]. In addition, improving HIV testing uptake will lead to better reach and coverage of subsequent HIV/AIDS care services because testing is considered a care sequence entry [29]. HIV testing programs may link participants to such services as self-help groups to maintain HIV-negative status or outpatient treatment for HIV-positive MWID. High rates of HIV testing are an important component for reaching the WHO 90-90-90 goals. We found that ever having been tested for HIV before this study was not associated with any of the needle–syringe sharing behaviors. This finding was in agreement with Do and colleagues’ study among people who injected drugs and had known and unknown HIV-positive status [3]. However, our finding contradicted those of Bergenstrom and colleagues from their northern Vietnam study, which showed that most people living with HIV/AIDS had adjusted their injection practices once they learned they were HIV positive [30]. That ever having been tested for HIV was not associated with needle–syringe sharing behaviors across studies may be explained by the low numbers of those who actually received the results of their HIV testing. Poor post-test counseling might also have contributed to this finding. Without appropriate counseling, MWID might have not been referred to support groups that provide psychological aid (e.g., raising awareness, peer-motivation) or access to free syringes and needles [31].

Our study showed that access to free syringes and needles would contribute to elimination of HIV transmission by infected MWID. Among the HIV-positive participants, those who received free clean needles and syringes were less likely to give their used needles and syringes to peers. Evidence from an eight-year project in Vietnam’s Lang Son province (bordering China), needle and syringe provision was one of the interventions that helped control the HIV epidemics among injection drug users [19]. Also, the World Health Organization (WHO)’s recommended needle and syringe programs as one of the nine service components in a comprehensive package to prevent and control HIV/AIDS among injection drug users. The remaining eight components include opioid substitution therapy and other drug dependence treatment; HIV testing and counseling; antiretroviral therapy; prevention and treatment of STIs; condom programs for injection drug users and their sexual partners; targeted information, education, and communication for injection drug users and their sexual partners; vaccination, diagnosis, and treatment of viral hepatitis; and prevention, diagnosis, and treatment of tuberculosis [32]. It is urgent to scale up free access to clean needles and syringes for the HIV-positive MWIDs in this region. As mentioned, the HIV testing rate among this group was low. Altogether, we recommend future HIV/AIDS interventions extensively offering free needles and syringes to the MWID in the region regardless of the HIV status.

In the northwest region, a low-resourced area, it is imperative to prioritize HIV testing and needle and syringe provision programs among the recommended WHO services components. Adding other service components will be contingent upon budget availability. At the time of the data collection, there were no methadone maintenance treatment (MMT) programs in Dien Bien and Lao Cai provinces. Previous studies showed MMT programs improved the referral and testing uptake among Vietnamese drug users and their partners [33], and ultimately these programs helped lower the healthcare costs in the long term [34]. Hence, policymakers and HIV/AIDS program implementers in the region may consider adding MMT programs as the next priority service component for the MWID.

The rate of MIWD who were sexually active in our study (42%) is within the range found in previous studies. In a study in Hai Phong, a northern city of Vietnam, 31% of the MWID were sexually active during the previous 3 months [35]. Go and colleagues found that 73% of the MWID in Bac Ninh, a northern province, were sexually active during the previous 6 months [36]. Also, one half to two thirds of the MWID in the border areas—Lang Son Province (Vietnam) and Ning Ming County (China)—reported being sexually active during the previous 6 months [37].

Going to a hotspot was an important factor because it was consistently associated with an increased likelihood of needle–syringe sharing behaviors in all subgroups (including MWID who had never been tested for HIV before this study and HIV-negative and HIV-positive MWID). Our finding corroborated the results of several studies that showed injecting drugs together with other users, injecting at hotspots, and injecting on the street or in public areas increased sharing of needles and syringes [1, 3]. Hotspots have been suggested to be an effective venue to deliver harm reduction programs (e.g., clean needle and syringe distribution and exchange) [38]. Therefore, policymakers and implementers of HIV/AIDS intervention programs might find greater impact if they target hotspots first.

We succeeded in recruiting a large sample of the MWID thanks to a well-organized preparation. Prior to designing the study, the research team spent multiple site visits learning about the MWID in the region. We established a productive rapport with the local authorities leading the HIV/AIDS response and with key peer educators (knowledgeable about numerous MWIDs and familiar with traveling across complex geographic areas.) Also, during these visits, the research team carefully arranged logistical preparation, i.e., identifying appropriate locations for the data collection that were the most accessible to potential study participants.

However, our study is not without limitations. Although the literature suggests that sexual and injection behaviors are strongly associated with HIV risk [3, 4], we could not account for the influence of sexual behaviors because our participants had a low rate of response to questions on sexual behaviors. Without accounting for this factor, our study may overestimate the associations between risk factors and HIV positivity or needle–syringe sharing behaviors. Previous studies also showed that HIV knowledge was an important predictor of HIV infection among people living in ethnic minority areas [22, 39]; however, this type of data was not available in our study. Future research may focus on exploring the link between HIV knowledge and risky injection behaviors among our MWID study population to better explain the complex HIV/AIDS epidemic in northwestern Vietnam. Of the three areas included in our study, Tuan Giao and Lao Cai City were at the same level of the census tract; Bat Xat was one level smaller. However, our previous site visits informed a considerable number of MWID in Bat Xat which was comparable with those in Tuan Giao and Lao Cai City. Therefore, it was reasonable to include Bat Xat in our analysis.

Chain referral sampling method, which is commonly known as one of the most practical and effective ways to reach hidden populations, [40, 41] helped us to recruit a large number of MIWD. Nevertheless, this sampling method might introduce some biases to representativeness and subsequent prevalence estimates in our study. We considered statistical adjustments using information on individual network size [42]; however, it was impractical to obtain the precise information. First, the actual network size was constantly changing during the time of data collection due to the anti-drug campaign. Second, estimation of network size and subsequent statistical adjustment require a documentation of individual linkages during the referral process. MWID deemed self-reporting their network information sensitive because they were afraid that law enforcement authorities would discover their connectedness with the drug user community and target other drug users for the anti-drug campaign at that time. Also, in our study, we asked all recruited participants to refer other potential participants (i.e., not just selected a few seeds in a large pool at the beginning and not limited the number of waves); thus, this mitigated the selection bias.

In addition, our results were subject to recall bias because most measures were based on self–report. However, to minimize this bias and better measure recent/current behaviors, we used short time frames (i.e., previous month for the frequency of injection drug use, previous week for going to a hotspot). Furthermore, we used different time frames for the same behaviors (e.g., needle–syringe sharing behaviors in the previous 6 months and the previous month) to aid recall and to enable response double checking. Lastly, because HIV infection status and needle–syringe sharing behaviors are sensitive topics, participants’ responses might be subject to social desirability bias.

## Conclusion

Our investigation of a subset of the MWID in the northwestern Vietnam found that these MWID of different ethnicities had different risks for HIV infection. To prevent and control an HIV/AIDS epidemic in this region, intervention programs and messages should be tailored to the culture and in the language of each ethnic group. While it is ideal to deliver all nine components of the WHO comprehensive package of HIV prevention, treatment, and care services, we recommend the top two priority services for the MWID in this resource-constrained region. These two services include enhanced HIV testing and needle and syringe provision. Because going to a hotspot was a salient factor associated with needle–syringe sharing behaviors, policymakers and implementers of HIV/AIDS intervention programs should first target such venues in order to achieve greater impact.

## Abbreviations

AIDS: Acquired immune deficiency syndrome
AOR: Adjusted odds ratio
CI: Confidence interval
HIV: Human immunodeficiency virus
IBBS: Integrated Biological and Behavioral Surveillance
MWID: Men who inject drugs
STI: Sexually transmitted infection
